# Three-Dimensional Modeling of the Pelvic Floor Support Systems of Subjects with and without Pelvic Organ Prolapse

**DOI:** 10.1155/2015/845985

**Published:** 2015-02-01

**Authors:** Shuang Ren, Bing Xie, Jianliu Wang, Qiguo Rong

**Affiliations:** ^1^College of Engineering, Peking University, Beijing 100871, China; ^2^Department of Obstetrics & Gynecology, Peking University People's Hospital, Peking University, Beijing 100871, China

## Abstract

The purpose of this study was to develop three-dimensional finite element models of the whole pelvic support systems of subjects with and without pelvic organ prolapse (POP) that can be used to simulate anterior and posterior wall prolapses. Magnetic resonance imaging was performed in one healthy female volunteer (55 years old, para 2) and one patient (56 years old, para 1) with anterior vaginal wall prolapse. Contours of the pelvic structures were traced by a trained gynecologist. Smoothing of the models was conducted and attachments among structures were established. Finite element models of the pelvic support system with anatomic details were established for both the healthy subject and the POP patient. The models include the uterus, vagina with cavity, cardinal and uterosacral ligaments, levator ani muscle, rectum, bladder, perineal body, pelvis, obturator internus, and coccygeal muscle. Major improvements were provided in the modeling of the supporting ligaments and the vagina with high anatomic precision. These anatomically accurate models can be expected to allow study of the mechanism of POP in more realistic physiological conditions. The resulting knowledge may provide theoretical help for clinical prevention and treatment of POP.

## 1. Introduction

Pelvic floor prolapse (POP) is defined as downward descent of the pelvic floor organs, anterior compartment (bladder and the urethra), uterus, vaginal cuff, and posterior compartment (rectum), resulting in protrusion of the vaginal wall, uterus, and/or vagina [1, 2]. The latest study showed that POP, when defined by symptoms, has a prevalence of 3–6%. When defined by a vaginal examination, however, this figure reaches 50% [3].

Although in a healthy woman the pelvic support system can hold the organs in normal positions, a subtle injury of this support system may lead to progressive POP. The support system of the uterus comprises a combined action of muscles and connective tissues [4]. Numerical simulations provide a tool with which to study pelvic function and the possible effects of support system defects [5] as these methods can be used to study the pathogenesis of POP by simulating various levels of impairment of the pelvic floor support system that cannot be performed clinically. Two-dimensional (2D) [6] and 3D [7] models of POP have been built to simulate prolapse of the anterior vaginal wall. These studies provided substantial clues for the diagnosis and treatment of POP, but there was oversimplification of apical ligament support in those studies. The vaginal cavity was also not considered when simulating cystocele (prolapse of the anterior vaginal wall) or rectocele (prolapse of the posterior wall). If the vagina model is created as a solid one, the anterior and posterior vaginal walls protrude together, so it is impossible to simulate the realistic situations of cystocele or rectocele. Chen et al. [7] solved this problem when simulating cystocele by combining the posterior vaginal wall and the rectum as the posterior compartment. However, the potential high stress on the lateral vaginal wall could not be observed. Additionally, the friction force caused by closing the vaginal cavity counteracts the descent of the vagina caused by increasing abdominal pressure [8]. Therefore, the vaginal cavity is of great significance when evaluating the mechanical behavior of POP.

Hence, two 3D models of vaginal support systems of subjects with and without POP that included a vaginal cavity were established for finite element analysis in this study. Attachments among the various structures were established using ANSYS software (ANSYS, Houston, TX, USA). The models based on the anatomy of these two subjects were evaluated with magnetic resonance imaging (MRI) and the 3D finite element models, and the results were compared.

## 2. Materials and Methods

We recruited one healthy female volunteer [55 years old, para 2; body mass index (BMI) 20.96 kg·m^−2^] and one patient (56 years old, para 1; BMI 27.89 kg·m^−2^) with anterior vaginal wall prolapse diagnosed based on clinical manifestations and the pelvic organ prolapse quantitation system (POP-Q). Both subjects signed informed consent for inclusion in this institutional review board-approved study. Each denied previous pelvic surgery and other contraindications to this procedure.

The two subjects underwent pelvic MRI using a 3.0-T (Discovery MR750 3.0 T; GE Healthcare, Milwaukee, WI, USA) and a 32-channel torso array coil wrapped around the abdomen and pelvis. T2-weighted fast recovery fast spin echo MRI was performed in the axial, sagittal, and coronal planes, respectively, with the following parameters: TR/TE 3000/102–108; field of view 26–28 cm; slice thickness 4 mm interleaved; gap 1 mm; acquisitions 2. Scan time was less than 10 min, and 90 continuous images were obtained.

The images in digital imaging and communication in medicine (DICOM) format were imported into the medical image processing software Mimics 10.01 (Materialise Inc., Leuven, Belgium) for 3D reconstruction. The images were manually segmented into anatomically significant components including pelvic bones, bladder, urethra, vagina, uterus, rectum, obturator internus, cardinal ligaments, uterosacral ligaments, and levator ani with the cooperation of a trained gynecologist and a senior experienced radiologist. The dimensions of the 3D models were then calculated and reconstructed in Mimics software. The 3D models were exported as STL files and imported into Geomagic Studio software (version 12.0; Geomagic, Inc., Morrisville, NC, USA) for smoothing, positioning, and modification without altering the structure for further finite element modeling.

Finally, the 3D models were exported from Geomagic Studio into IGES format files, which were imported into ANSYS software (version 14.0). Connections between the various structures were established by Boolean operations.

## 3. Results

**Figure 1:**
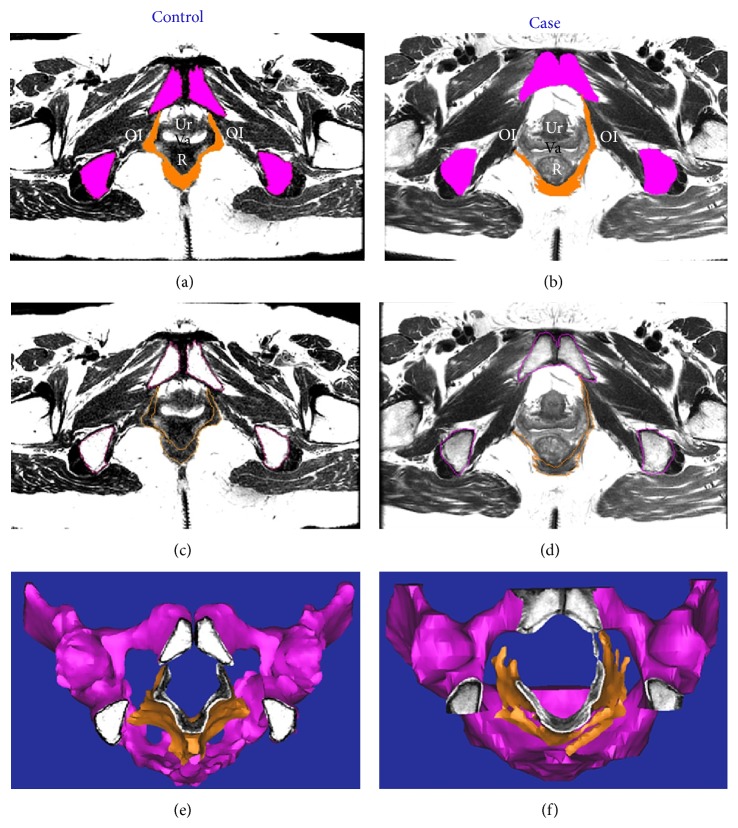
Axis magnetic resonance imaging (MRI) of a subject without ((a), (c)) and a patient with ((b), (d)) pelvic organ prolapse (POP). Top panel shows regions of the pelvis (magenta) and the levator ani muscle (orange). Middle panel shows outlines of the structures. Bottom panel shows a three-dimensional (3D) model on MRI. Note that the levator ani muscle of the control subject attaches to the pubic bones on both sides, whereas that of the POP patient has defects on the right side. Ur: urethra; Va: vagina; R: rectum; OI: obturator internus.

**Figure 2:**
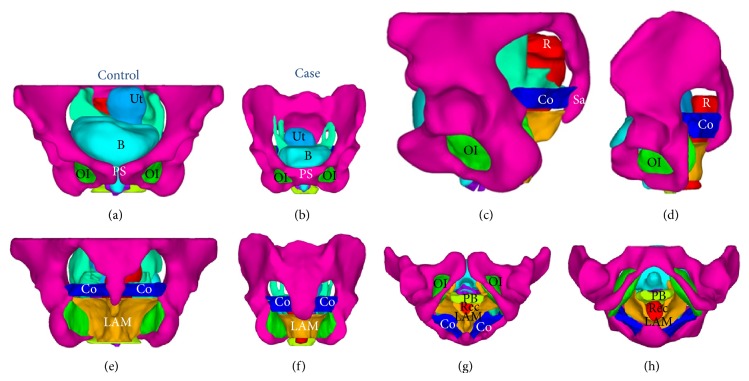
3D models of the pelvic floor of a subject without POP (top panel) and a patient with POP (bottom panel). Shown are the pelvis (magenta), uterus (sky blue), bladder and urethra (turquoise), cardinal and uterosacral ligaments complex (spring green), levator ani muscle (orange), rectum (red), coccygeus (blue), obturator internus (lime), perineal body (green-yellow), and vagina (dark violet). (a), (b): front view. (c), (d): left view. (e), (f): back view. (g), (h): dorsal lithotomy view. Ut: uterus; B: bladder; Co: coccygeus; LAM: levator ani muscle; R: rectum; OI: obturator internus; Sa: sacrococcyx; PS: pubic symphysis.

Axial MRI scans and 3D models of the levator ani muscle and pelvis are shown in Figure 1. The levator ani muscle of the healthy woman is thicker and is V-shaped, whereas that of the POP patient is thinner and is U-shaped. Some defects are apparent on the right side of the levator ani muscle in the POP patient, where the muscle is attached to the pubic bone. 3D models of the pelvic floor of subjects without and with POP that include 10 components are shown in Figure 2.

**Figure 3:**
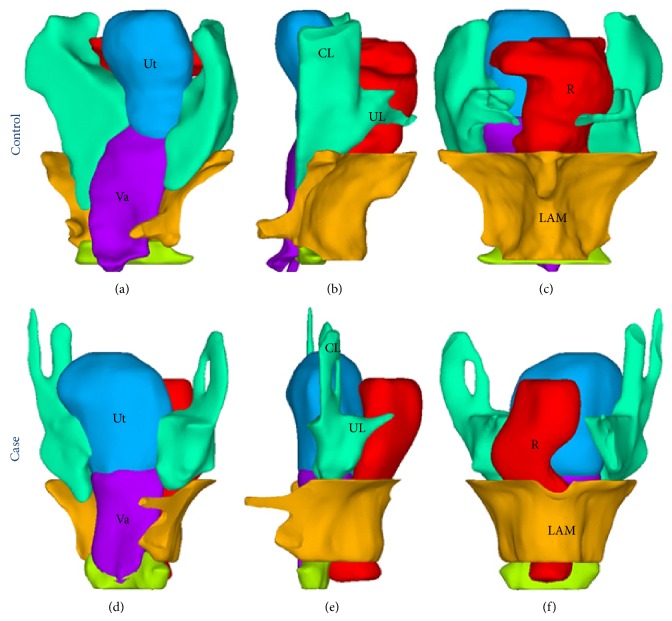
Uterine and vaginal support system. Apical support is provided by the CL and UL. Lateral support is provided by the LAM, which wraps around the vagina laterally. Distal support comes from the PB, which attaches to the vagina and rectum, and the puboperinealis ramus of the LAM, which is inserted into the PB. CL: cardinal ligament (spring green); UL: uterosacral ligament (spring green); R: rectum (red); LAM: levator ani muscle (orange); Va: vagina (dark violet); perineal body (green-yellow).

**Figure 4:**
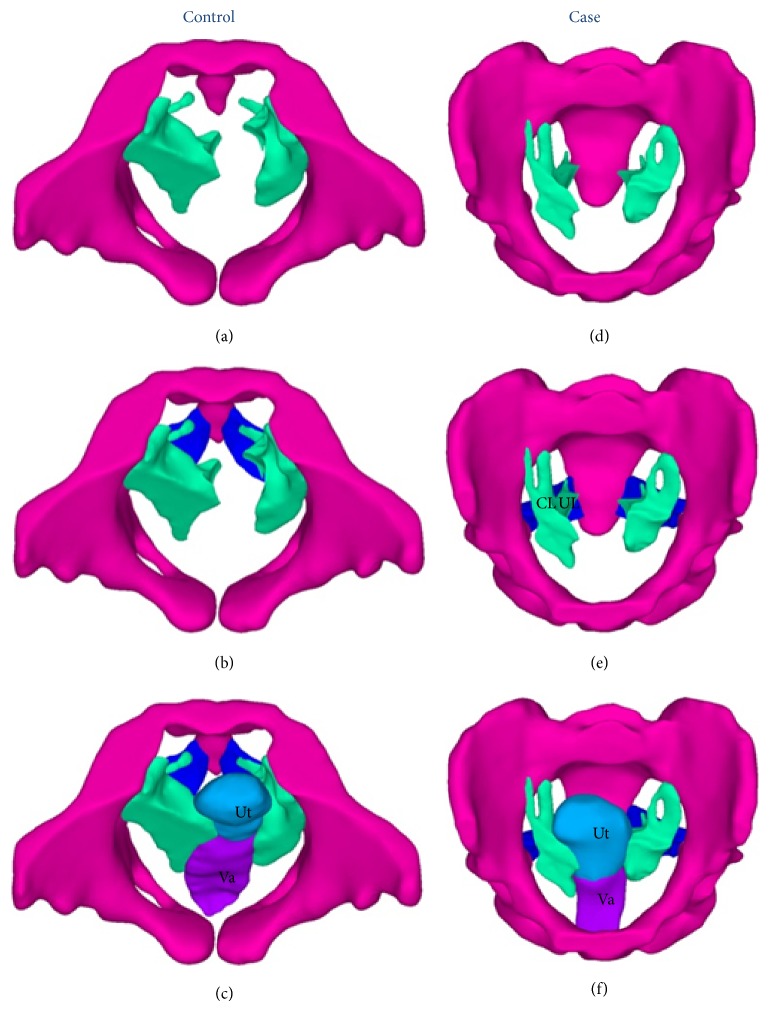
Attachment of the ligaments to the pelvic floor. Ligament complex attaches to the pelvic wall superiorly and to the coccygeus posteriorly. Coccygeus attaches to the pelvic wall on both sides. Ligament complex attaches to the cervix inwardly. The cervix in our model is fused with the uterus.

Regarding the attachments that supply apical support, the cardinal ligaments of the volunteer subject were much thicker than those in the POP patient (Figure 3). Also, there is some loss of attachment to the lateral pelvic wall in the POP patient. The 3D representation (Figure 4) shows that the attachment of uterosacral ligaments to the sacrum in the POP patient is thinner than that in the volunteer. Cardinal and uterosacral ligaments were combined because they were overlapped around the cervix [9] and had similar mechanical properties. Similarly, the cardinal and uterosacral ligaments were attached to the upper third of the vagina. The bottom of the uterus was attached to the top of the vagina. The ligament complex was attached to the cervix inwardly, to the apex pelvic wall (the ilium) upwardly, and to the posterior pelvic wall by its attachment to the coccygeus dorsally (Figure 4).

**Figure 5:**
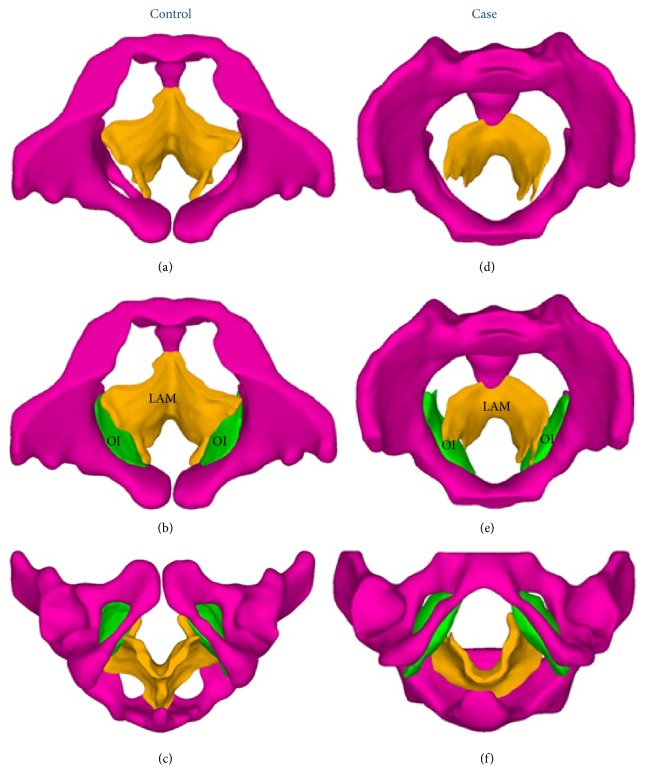
Attachment of the levator ani muscle with the pelvis. The LAM attaches to the coccygeus posteriorly and laterally to the tendinous arch of the pelvic fascia (ATFP), which lies on the OI. Note that in our model the LAM attaches to the OI laterally. The OI attaches to the obturator foramen. LAM: levator ani muscle (orange); OI: obturator internus (lime); pelvis (magenta).

For attachments of structures that provide bilateral support, the levator ani muscle arises from the arcus tendineus levator ani, which overlies the obturator internus [10] and attaches to the pelvic wall on each side. In our model, the levator ani muscle was attached to the internal obturator muscle bilaterally [10], to the coccyx posteriorly, and to the pubic bones anteriorly (Figure 5). The arcus tendineus levator ani was omitted for simplicity. The rectum leaned against the levator ani muscle. The bilateral ends of the levator ani muscle arose from the pubic bones and passed behind the rectum, forming a hammock-like arrangement (Figure 5) [11].

Regarding attachments of the distal vagina, it is mainly the perineal body that provides distal support of the vagina. The perineal body is a fibromuscular mass between the urogenital and anal triangles of the perineum [12]. It is bordered anteriorly by the posterior vaginal wall, posteriorly by the anterior anorectal wall, and laterally by the ischial rami. Along its upper lateral boundaries, the perineal body is attached to the pubococcygeus, pubovaginalis, and puborectalis of the levator ani muscle group [12]. Therefore, in our model, the perineal body was attached anteriorly to the posterior vaginal wall, posteriorly to the anterior anorectal wall, and laterally to the levator ani muscle (Figure 3).

The attachments of the POP patient were the same with those in the volunteer. The internal obturator muscles and the coccygeus were attached to the pelvic wall (Figure 2).

**Figure 6:**
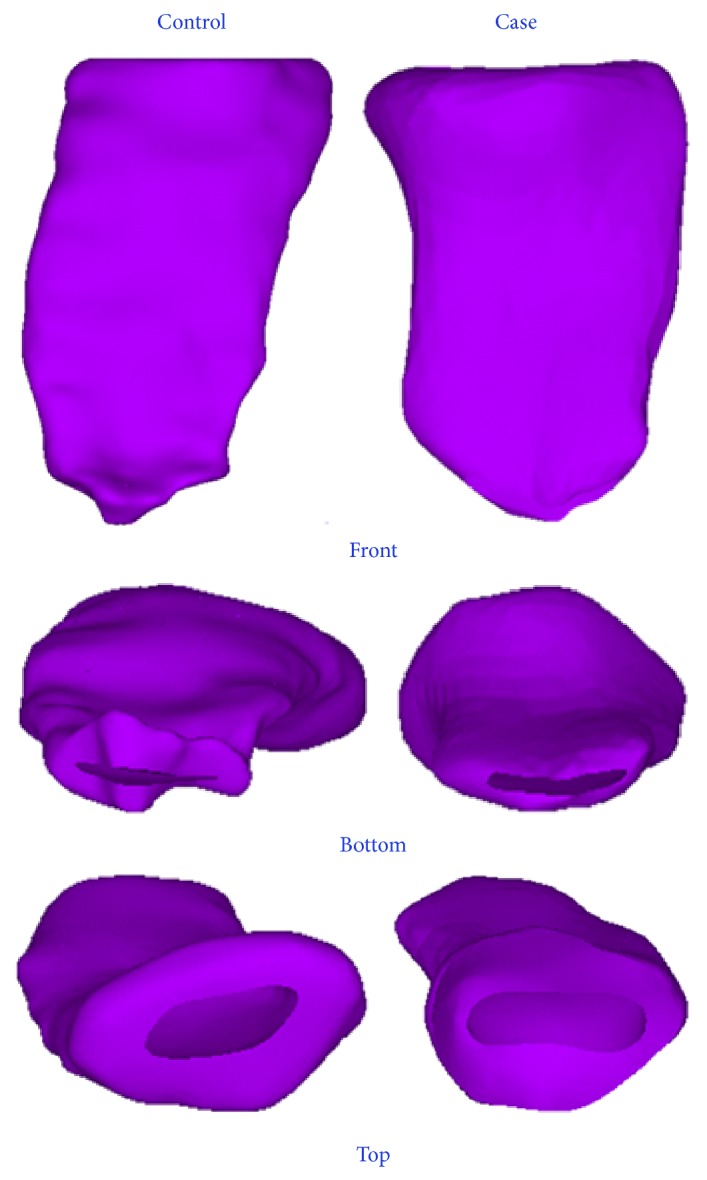
Vaginal lumens. Left: subject without POP. Right: patient with POP. The vaginal lumen is a thin canal that runs throughout the vagina. Axenic coupling medium was inserted into the vagina before MRI scanning, making the vaginal cavity identifiable. The modeling of the vaginas was psychologically not realistic, so these models were used only for demonstration and should be modified in the finite element calculations.

The vagina is an elastic muscular canal (Figure 6). The model in this study was built by subtracting Boolean calculations of the solid vagina structure and the internal cavity.

**Figure 7:**
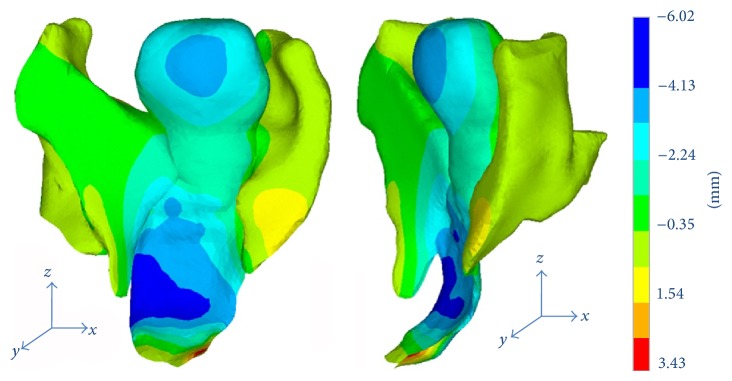
The vertical displacement of the pelvic organs without POP under a pressure of 0.5 KPa. The tendency of vagina deformation was consistent with the clinical observations. The maximum descent occurred in the intermediate zone of the vagina.

Simulations with these two FE models were performed to validate the feasibility and accuracy. The preliminary results showed that tissue damage and loss of anatomic integrity were the trigger for POP. The support structures of the pelvic floor moved downward and backward when anterior vaginal wall prolapse occurred, as shown in Figure 7. Damage of ligaments and/or vagina, along with the raised intra-abdominal pressure, can lead to anterior vaginal wall prolapse. Concurrence of the ligaments and anterior vaginal wall impairment resulted in the severest prolapse, with the largest displacement in the lower intermediate region of the vagina. The larger the damage of the supporting structures, the severer the prolapse. The von Mises stress concentrated in the anterior vaginal wall and its attachments to the support structures, where the lateral sides of the anterior vaginal wall connected with the cardinal and uterosacral ligaments and paracolpium.

The simulations were only a preliminary work. The constitutive models of relevant tissues, the loading and boundary conditions, and the interactions among different tissues and structures, and so forth, all these have to be critically validated before application in clinical studies.

## 4. Discussion

In this study, the levator ani muscle, cardinal ligament, and uterosacral ligament of subjects with and without POP were compared using MR images and 3D models. Details of the finite element analysis of attachments of this composite support system for the vagina are presented.

The POP patient model revealed defects of the attachments on the left side and loss of muscle fibers of the levator ani muscle. In 2006, Chen et al. [13] developed a 3D model of the female levator ani muscle with unilateral right defects. A method to quantify the cross-sectional area of the pubic portion of the levator ani muscle has also been devised. The levator ani muscle damage usually appears more often in the pubic portion than in the iliococcygeal portion [14], which corresponds to the area of damage in our study. For simplification in this study, we did not separate the levator ani muscle into subdivisions [10] (puboanal, puboperineal, pubovaginal, puborectal, and iliococcygeal). Separating the levator ani into multiple portions presents challenges as there are small areas where subdivisions overlap [10, 13]. Further refinements in MRI and advances in the finite element analysis may allow more accurate separation in the future.

The cardinal ligaments were found to be thinner in the POP patient in our study than in the volunteer. Ramanah et al. [9] developed 3D models of the cardinal and uterosacral ligaments. They established important structural specifics of the cardinal ligament's attachments and its relation with the uterosacral ligament. That research group found that the cardinal ligament has an origin at either the anterior trunk of the internal iliac artery (35%) or the upper border of the greater sciatic foramen (65%). The cardinal and uterosacral ligaments have a common region of insertion at the cervix and/or the upper vagina [9]. Therefore, the cardinal ligament and the uterosacral ligament were merged as one entirety in our model.

The perineal body, known as the anchor of the pelvis [15], provides support to the lower vagina. Larson et al. [15] built a 3D model of the pelvic floor to analyze the complex anatomy of the perineal body in living women with normal pelvic support. Three distinct regions were suggested for the perineal body: a superficial region at the level of the vestibular bulb; a midregion at the proximal end of the superficial transverse perineal muscle; and a deep region at the level of the midurethra and puborectalis muscle. The puborectalis muscle loops behind the rectum, likely helping to suspend the perineal body from the pubic bone [15, 16]. In our model, the perineal body attached to the levator ani muscle laterally, to the vagina anteriorly, and to the rectum posteriorly.

The vagina is an elastic, muscular canal with a soft, flexible lining. The anterior and posterior vaginal walls share common attachments with the ligaments [17]. In the upper one-third of the vagina, the vaginal walls are connected laterally and dorsally by the cardinal and vaginal portion of the uterosacral ligament [17]. The lower one-third of the vagina is fused with the perineal body [17]. In the midvagina, the wall is connected to sheets of endopelvic fascia that merge with the arcus tendineus fascia pelvis [17]. When the levator ani muscle contracts, it squeezes the midurethra, distal vagina, and rectum against the pubic bone distally [18], preventing the vaginal wall from being exposed to atmospheric pressure [17]. This action closes the vaginal cavity, shutting off the urogenital hiatus through which prolapse occurs. Friction force caused by contact of the anterior vagina wall with the posterior wall may counteract increases in intra-abdominal pressure, thereby reducing tension on the support ligaments [8].

Several studies have used numerical simulation to address POP [5–7, 19–23]. 2D [6] and 3D [7] finite element models of cystocele have been developed to examine how impairment of the levator ani muscle and/or the ligaments are related to cystocele. Various levels of impairment of the ligament or/and the levator ani muscle were simulated. However, ligaments were usually simplified as springs [6, 7]. For a finite element model of the female pelvic floor, including the levator ani muscle with anisotropic viscohyperelastic behavior and the fetal head, it took about 3.5 hours to complete a single simulation in a computational environment involving a Pentium dual core 3.0 GHz CPU with 3 G RAM and running Windows XP [23].

Finite element analysis of soft tissues is still a challenge. Contacts between different tissues increase the modeling complexity. Therefore, the models derived from Mimics software were smoothed and modified slightly in the Geomagic software. Attachments were built by sharing contact faces through Boolean calculations in ANSYS software. Attachments of the model of the POP patient were established in the same way, while loss of the tissue or defects of the attachments can be simulated by weakening the material properties in specific regions. Boundary conditions play a key role in the simulation. Further study is needed to define realistic boundary and loading conditions.

Finite element models of cystocele in previous studies usually oversimplified the structure of the ligaments and the vagina. The vagina was modeled as a single anterior vaginal wall. The present study tried to develop finite element models for the complete support system of the uterus and vagina. The vaginal cavities were considered in both models. The 3D finite element models can be applied to studying the mechanism of POP and provide help for clinical prevention and treatment. The main improvements of this study lie in the modeling of the entire pelvic floor support system and establishing more realistic contacts between the various structures.

There are some limitations in this study. The model lacks paravaginal supports, which could be simulated by membrane elements in a fan-shaped pattern originating from the ventral insertion of the arcus tendineus fascia pelvis with insertion in the lateral aspect of the vagina, as described by Chen et al. [7].

## 5. Conclusion

This study focused on 3D models of the vagina and uterus and their support system in subjects with and without POP. The vaginal cavity was considered for the first time in finite element modeling of the pelvic floor system. The preliminary simulation results were consistent with the clinical observations.
